# Evaluation of direct and indirect effects of seasonal malaria chemoprevention in Mali

**DOI:** 10.1038/s41598-018-26474-6

**Published:** 2018-05-25

**Authors:** Thomas Druetz

**Affiliations:** 1 Center for Applied Malaria Research and Evaluation, Department of Tropical Medicine, Tulane University, New Orleans, USA; 2 Department of Social and Preventive Medicine, School of Public Health, University of Montreal, Montreal, Canada

## Abstract

Randomized controlled trials have established that seasonal malaria chemoprevention (SMC) in children is a promising strategy to reduce malaria transmission in Sahelian West Africa. This strategy was recently introduced in a dozen countries, and about 12 million children received SMC in 2016. However, evidence on SMC effectiveness under routine programme conditions is sparse. We aim to measure the effects of the nationwide SMC programme in Mali on the prevalence of malaria and anemia in children 6–59 months. We used data from the 2015 nationally representative malaria indicator survey. A post-test only with non-randomized control group study was designed. We fitted a generalized structural equation model that controlled for potential bias on observed and non-observed variables (endogenous treatment effect model). Having received SMC reduced by 44% (95% CI [0.39–0.49]) the risk of having a positive rapid diagnostic test for malaria. In addition, the programme indirectly reduced by 18% the risk of moderate-to-severe anemia (95% CI [0.15–0.21]). SMC in Mali has substantial protective effects under routine nationwide programme conditions. Endogenous treatment effects analyses can contribute to rigorously measuring the effectiveness of health programmes and to bridging a widening gap in evaluation methods to measure progress towards achieving malaria elimination.

## Introduction

Seasonal malaria chemoprevention (SMC) is a promising strategy to reduce the burden attributable to malaria in children under five. It consists of the repeated administration of a long-acting antimalarial drug regimen in areas where malaria transmission is highly seasonal^1,2^. Drug administration usually takes place once per month during the transmission season, for a total of three or four cycles per year depending on the length of the rainy season. This strategy typically targets all children 3–59 months, and combines therapeutic and prophylactic effects. The drug regimen recommended in West African countries is one single-dose of sulfadoxine-pyrimethamine (SP) and three daily doses of amodiaquine (3AQ)^1,3^.

SMC efficacy has been demonstrated in several randomized controlled trials^4–10^. A meta-analysis, pooling data from seven trials, showed a large and significant reduction in the incidence of clinical malaria (82%, 95% CI 75–87) over the intervention period^11^. One particular trial showed that SMC reduces the prevalence of malaria infection by 85% (95% CI 73–92) during the transmission season^7^. Trials also suggested a beneficial effect on anemia^7,8^. Based on this evidence, the WHO has been recommending SMC since 2012, and it has been introduced in a dozen Sahelian or sub-Sahelian countries^12,13^. Thanks to this rapid scale-up, about 12 million children received SMC in 2016^14^.

Outside of experimental contexts, little is known about the effectiveness of routinely implemented SMC programmes. A pre-post study with a non-randomized control group conducted in Burkina Faso showed that after the first cycle of SMC, the prevalence of malaria parasitaemia was reduced by 62% (95% CI 48–71)^15^. An ecological study conducted in Mali with a similar quasi-experimental design found that SMC reduced the odds of malaria infection at the district level by two thirds (OR 0.35, 95% CI 0.19–0.66) at the end of the transmission season^16^. Unfortunately, these studies were limited to small study areas and have limited external validity. To date, there have been no evaluations of SMC impacts at the regional, national or sub-national levels using routine data or nationally representative surveys.

We aim to assess the effectiveness of SMC at reducing the prevalence of malaria and anemia in children 6–59 months, using data from the 2015 malaria indicator surveys (MIS) in Mali. Our intention is also to illustrate that nationally representative surveys, such as the MIS or the demographic and health surveys, can provide a rich resource for natural experiments and rigorous evaluation designs.

## Results

A total of 5,960 children aged 6–59 months were included in the MIS survey. Their characteristics are summarized in Table 1. The caregivers of 2,666 of these children (45%) reported that they had received an SMC treatment in 2015. SMC cards were available and checked for 1,951 children. Self-reported exposure to SMC was not statistically significantly associated with any of the children’s characteristics, i.e., sex, age, or use of bed nets. Exposure to SMC was statistically significantly associated with variables at higher levels: mother’s education, household’s size, socio-economic status, religion, number of bed nets, and cluster altitude and region.

**Table 1:** Population characteristics.

	Received SMC treatment		Test statistic (p-value)
	No	Yes	
Number	3,294	2,666	
Female	50%	48%	χ^2^ = 2.83 (0.092)
Mean age in months (SD)	32 (16)	32 (16)	t = −1.52 (0.127)
Slept under a bed net the night before	73%	74%	χ^2^ = 1.47 (0.225)
Mothers’ education			χ^2^ = 46.75 (<0.001)
None	77%	80%	
Primary	11%	13%	
Secondary or higher	12%	7%	
Ethnic group			χ^2^ = 3.76 (0.152)
Bambara	31%	32%	
Peulh/Toucouleur	17%	15%	
Other	52%	53%	
SES			χ^2^ = 355.21 (<0.001)
Poorest	20%	22%	
Poorer	20%	26%	
Medium	18%	26%	
Richer	18%	20%	
Richest	24%	6%	
Size of the household			χ^2^ = 20.57 (<0.001)
1–5	13%	11%	
6–10	36%	33%	
≥10	51%	56%	
Muslim	93%	90%	χ^2^ = 14.15 (<0.001)
Mean number of bed nets (SD)	4.29 (2.2)	4.57 (2.1)	t = −5.04 (<0.001)
Cluster altitude in meters (SD)	322 (82)	291 (89)	t = 14.06 (<0.001)

SD standard deviation; SES socio-economic status; SMC seasonal malaria chemoprevention.

### Model selection

Three different types of models were used to measure the effects of SMC on malaria: multiple regression (Model I), multiple regression with endogenous treatment (Model II), and generalized structural equation models with endogenous treatment (Modell III). These models were fitted for the two primary study outcomes, i.e., RDT- and microscopy-confirmed malaria. Results are shown in Tables 2 and 3, respectively. For both outcomes, the Wald test of independent equations in Model II suggests that errors for the treatment and the outcome are correlated (Chi^2^ = 22.52 and Chi^2^ = 16.57, respectively, and both have *P*-values < 0.001). Therefore, endogenous Models II and III were preferred over Model I since they control for potential selection on unobservables (“treatment selection bias”). As a GSEM, Model III has the additional advantage of allowing mediation relationships to be included in the analysis, and therefore to measure SMC direct and indirect effects on anemia. For this reason, Model III was retained as the final model for the analysis.

**Table 2:** Models of the effects of SMC on RDT-confirmed malaria.

	Model I Multiple regression	Model II Multiple regression with endogenous treatment	Model III GSEM with endogenous treatment
	RR (95% CI)	RR (95% CI)	RR (95% CI)
Outcome: RDT			
SMC	0.60 [0.55–0.64]	0.56 [0.51–0.61]	0.56 [0.51–0.61]
Age (in months)	1.05 [1.04–1.07]	1.05 [1.04–1.07]	1.06 [1.05–1.08]
Region			
Kayes (ref.)	1	1	1
Koulikoro	1.52 [1.30–1.76]	1.50 [1.29–1.74]	1.54 [1.31–1.82]
Sikasso	1.89 [1.64–2.17]	1.87 [1.62–2.15]	2.02 [1.73–2.36]
Segou	1.33 [1.14–1.55]	1.32 [1.13–1.54]	1.35 [1.14–1.59]
Mopti	1.92 [1.67–2.21]	1.89 [1.64–2.18]	2.05 [1.76–2.39]
Bamako	0.43 [0.26–0.73]	0.42 [0.25–0.71]	0.44 [0.26–0.75]
SES			
Poorest (ref.)	1		
Poorer	0.97 [0.90–1.06]	0.98 [0.90–1.06]	0.97 [0.88–1.07]
Middle	0.81 [0.73–0.89]	0.81 [0.73–0.89]	0.78 [0.70–0.87]
Richer	0.54 [0.47–0.61]	0.54 [0.47–0.61]	0.50 [0.43–0.57]
Richest	0.12 [0.08–0.18]	0.12 [0.08–0.18]	0.11 [0.07–0.16]
Ethnic			
Bambara	1	1	1
Peuhl/Toucouleur	0.80 [0.72–0.90]	0.80 [0.72–0.90]	0.77 [0.67–0.87]
Other	0.98 [0.90–1.07]	0.98 [0.90–1.07]	0.97 [0.88–1.07]
Mother’s education			
None	1	1	1
Primary	0.92 [0.80–1.04]	0.92 [0.81–1.05]	0.91 [0.79–1.06]
Secondary or higher	0.75 [0.60–0.93]	0.75 [0.60–0.93]	0.71 [0.56–0.90]

Wald test of no correlation between outcome and treatment equations (Model II): χ^2^ = 15.56 (<0.001).

RDT rapid diagnostic test; RR risk ratio; CI confidence interval; SMC seasonal malaria chemoprevention; SES socioeconomic status; GSEM generalized structural equation model.

**Table 3:** Models of the effects of SMC on microscopy-confirmed malaria.

	Model I Multiple regression	Model II Multiple regression with endogenous treatment	Model III GSEM with endogenous treatment
	RR (95% CI)	RR (95% CI)	RR (95% CI)
Outcome: Microscopy			
SMC	0.68 [0.64–0.73]	0.67 [0.63–0.72]	0.65 [0.60–0.71]
Age (in months)	1.03 [1.02–1.04]	1.03 [1.02–1.04]	1.03 [1.02–1.04]
Region			
Kayes (ref.)	1	1	1
Koulikoro	1.21 [1.06–1.38]	1.21 [1.06–1.37]	1.22 [1.06–1.41]
Sikasso	1.38 [1.22–1.55]	1.37 [1.22–1.55]	1.42 [1.24–1.62]
Segou	1.17 [1.03–1.33]	1.17 [1.03–1.33]	1.18 [1.02–1.36]
Mopti	1.72 [1.53–1.93]	1.71 [1.53–1.92]	1.84 [1.62–2.09]
Bamako	0.36 [0.25–0.51]	0.35 [0.25–0.51]	0.34 [0.24–0.50]
SES			
Poorest (ref.)	1	1	1
Poorer	1.01 [0.94–1.10]	1.01 [0.94–1.10]	1.02 [0.93–1.12]
Middle	0.81 [0.74–0.89]	0.81 [0.74–0.89]	0.79 [0.71–0.87]
Richer	0.65 [0.58–0.73]	0.65 [0.58–0.73]	0.62 [0.55–0.70]
Richest	0.40 [0.31–0.51]	0.40 [0.31–0.51]	0.37 [0.29–0.50]
Ethnic			
Bambara	1	1	1
Peuhl/Toucouleur	0.75 [0.67–0.84]	0.75 [0.67–0.84]	0.72 [0.64–0.82]
Other	1.01 [0.93–1.09]	1.01 [0.93–1.09]	1.01 [0.92–1.11]
Mother’s education			
None	1	1	1
Primary	0.87 [0.77–0.99]	0.88 [0.77–0.99]	0.85 [0.75–0.98]
Secondary or higher	0.76 [0.63–0.92]	0.76 [0.63–0.92]	0.75 [0.61–0.92]

Wald test of no correlation between outcome and treatment equations (Model II): χ^2^ = 10.25 (0.001).

RR risk ratio; CI confidence interval; SMC seasonal malaria chemoprevention; SES socioeconomic status; GSEM generalized structural equation model.

### Treatment effects on malaria

Children who received SMC had a lower risk ratio (RR = 0.556, 95% CI [0.51–0.61]) for RDT-confirmed malaria compared to those who did not, after adjustment for covariates and for endogenous treatment effects. The treatment effect was slightly smaller for microscopy-confirmed malaria, a risk reduction of 35% (RR = 0.652; 95% CI [0.60–0.71]). Children were at significantly higher risk of malaria if they were older and if they lived in regions other than Kayes (Koulikoro, Sikasso, Segou or Mopti). They were at significantly lower risk of malaria if they lived in the capital area, if their household had a higher SES, were Peulh or Toucouleur, or if their mother received secondary education (or higher). Covariates’ influence on the outcomes were very similar regardless of which test was used to detect parasitaemia, and was always in the anticipated direction.

### Treatment effects on anemia

**Figure 1:**
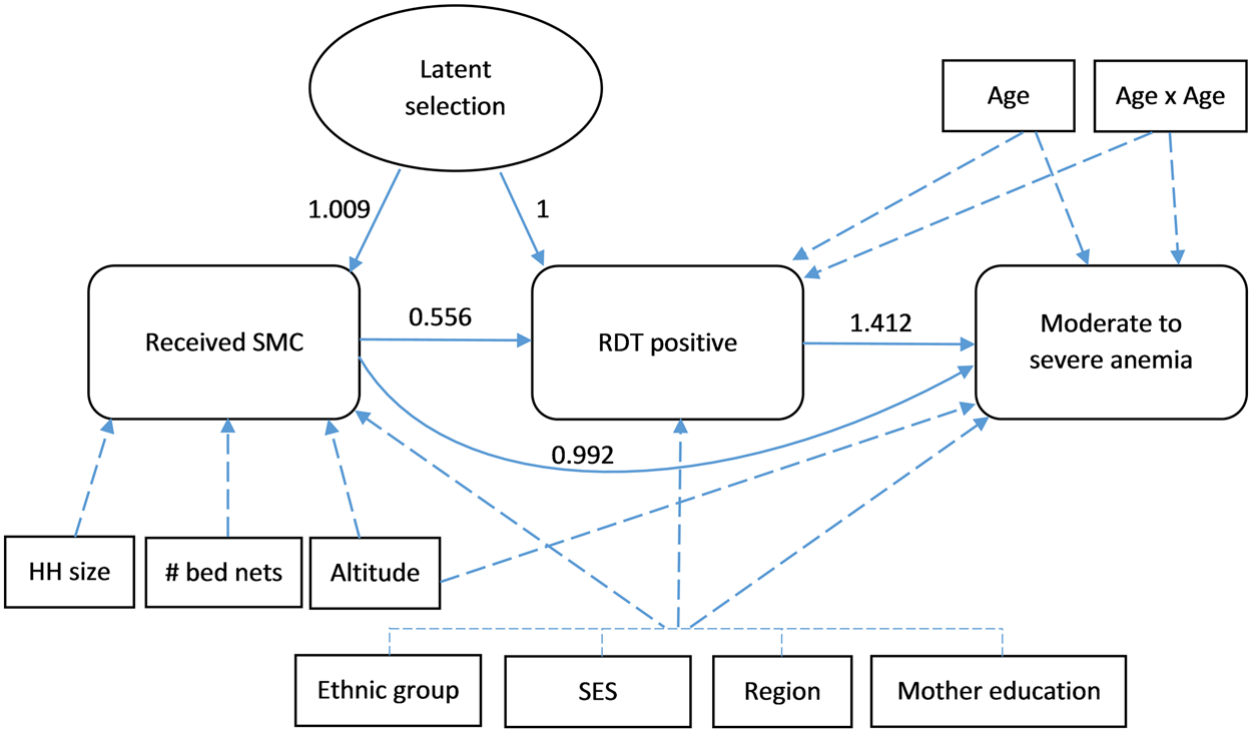
Flow chart of the SMC direct effects on RDT-confirmed malaria and the SMC indirect effects on moderate-to-severe anemia. Effect estimates were obtained by fitting a generalized structural equation model. Solid dashes link the outcomes and the latent variable between them. Long dashes link covariates to the outcomes. SES socioeconomic status; HH household; RDT rapid diagnostic test; SMC seasonal malaria chemoprevention; #number.

The path diagram of the mediated effects of SMC is shown in Fig. 1. As expected, the placebo test revealed the absence of a significant direct effect of SMC on moderate to severe anemia (RR = 0.992; 95% CI [0.95–1.03]). However, children whose RDT was positive were 41% (RR = 1.412, 95% CI [1.36–1.47]) more likely to be anemic compared to those whose RDT was negative, after adjustment for covariates. Therefore, indirect effects on anemia were computed using the product of coefficient method^17^. Children who received SMC had an 18% reduction in risk of anemia compared to those who did not (RR = 0.817; 95% CI [0.79–0.85]).

## Discussion

### Limits and strengths

Post-test only with control group designs are subject to selection bias if exposed individuals differ from unexposed individuals in characteristics correlated with exposure and outcomes variables. This study is based on a natural experiment – we had no control over the process through which survey participants were exposed or not exposed to SMC. As such, it is plausible that participants differ according to observed or unobserved characteristics. Several measures were taken to reduce the risk of a selection bias and increase the internal validity of the impact evaluation. First, data was collected using a two-stage cluster sampling and is representative of the national population, except for the regions of Gao, Tombouctou and Kidal. Second, we used multiple regression to control for a set of observed and potentially confounding factors. Third, we fitted endogenous treatment effects models to control for unobserved residual heterogeneity between the treatment and control groups.

Another strength of this study is the use of a theoretical replication strategy^18^. Indeed, the same hypothesis was tested on three different outcome indicators, representing different components of the chain of effects^19^. Results were always in agreement with those expected. Furthermore, a placebo test with a fake outcome was realized by differentiating SMC’s direct and indirect effects on anaemia. These tests increase the robustness of the results and our capacity to attribute the effects to the intervention^20,21^.

Analyses were intentionally conservative; bilateral statistical tests were used, and we evaluated the effects of being exposed to at least one SMC cycle, while the theory of intervention entails 4 cycles. Furthermore, in Mali, SMC is coupled with a test and treat strategy – if the RDT is positive, children receive Artemisinin-based combination therapy (ACTs), and if it is negative, children receive SP-AQ. Children were included in the analysis even if they had received ACTs rather than SMC. This could have diluted the estimate of SMC treatment effect.

The risk of recall bias cannot be excluded, since MIS implies a retrospective recording of practices and events. When possible, self-reported information was cross-validated by direct observation. Another limit is that it was not possible to assess SMC impact depending on the adherence level, because this information was not collected during the MIS. Also, although coverage was already high in 2015, SMC was only introduced nation-wide in 2016. So survey participants could have been unexposed to SMC in 2015 because they lived in an area that was not targeted. Finally, the fitted GSEM has several assumptions, including that causal effects are unidirectional (model is recursive) and that it is fully parametric. Because traditional tests are unavailable for GSEM, goodness-of-fit was indirectly checked by comparing the GSEM to a regression with endogenous treatment effects model.

### SMC impact evaluation

This study shows that exposure to at least one cycle of the 2015 SMC campaign in Mali had positive and significant (clinically and statistically) effects on malaria and anemia in children aged 6–59 months. The risk of RDT-confirmed malaria was reduced by 44%. With a national prevalence over 35% in children under five during high transmission season^22^, SMC has the potential for major public health impact in terms of morbidity, mortality and use of health services attributable to malaria^16^. However, the effect measured in the present study was lower than the 62% reduction in period prevalence measured in Burkina Faso during a routinely implemented SMC campaign^15^. Several factors may have contributed to a lower treatment effect in Mali compared to the one measured in Burkina Faso, including resistance to SP and/or AQ, lower adherence levels among children, a lower validity of the instruments, and the coupling of a test and treat strategy with SMC. In addition, contrary to the MIS in Mali, the study in Burkina Faso was not nationally representative of the population, but was conducted in a small area of a single district. The SMC treatment effect for microscopy-confirmed malaria, i.e. a reduction of 35%, was more modest than for RDT-confirmed malaria. This is potentially due to the lower sensitivity of microscopy vs. RDTs^23,24^. The lower capacity of microscopy to detect positives in both groups likely diluted the treatment effect. Results for both outcomes are presented here so readers can appreciate and compare them. A previous study conducted in two Malian districts suggested a larger effect; SMC was associated with a 65% reduction in the odds ratio of being infected by *Plasmodium* (measured by microscopy). However, this larger effect can partly be attributable to the fact that odds ratios overestimate risk ratios^25^. Also, the previous study was conducted after the full SMC campaign, while prevalence in the present study was measured during a SMC campaign.

However, even if we conservatively estimate that SMC effectiveness at reducing prevalence is 35–44%, results of such magnitude at the national level are promising for a routinely implemented programme. While approximately 45% of the children belonged to the treatment group in this nationwide natural experiment, it is important to sustain the efforts to increase SMC coverage in Mali.

The study also showed that SMC has no direct effect on moderate to severe anemia, but indirectly reduced its likelihood by 18%. The indirect effects on anemia were smaller than the direct effects on malaria parasitaemia. This is consistent with the fact that only a fraction of the risk of anemia is attributable to malaria, and that this fraction varies depending on the cut-off used to define anemia^26,27^. In this study, moderate to severe anaemia in children under five was defined as haemoglobin concentration <10 g/dl, as per WHO guidelines^28^. However, many SMC impact studies use a cut-off of 8 g/dl instead^11,29^. Had this lower cut-off been used here, SMC indirect effect on anaemia would have been larger (RR: 0.52, 95% CI [0.46–0.58]), and comparable to the results obtained from previous studies conducted in high transmission settings^7,8,16^.

Finally, this study also contributes to the development and promotion of rigorous evaluation designs using nationally representative surveys, such as the MIS or the demographic health surveys. While these surveys are commonly used for descriptive purposes and comparisons across countries or over time^30^, they can also lead to natural experiments designed to evaluate the impacts of new health interventions or policies^31–33^. This study illustrates how endogenous treatment effects models can be fitted with sectional survey data to evaluate the effectiveness of interventions using a post-test only (with control group) design. Despite its pitfalls (see limits above), this type of design is useful when there is no pre-test observation, or when pre-test data was collected too long before the intervention to constitute an adequate counterfactual^21^. This is often the case with MIS or nationally representative surveys, usually repeated every three years or so only.

The need to measure the impacts using rigorous quasi-experimental studies is particularly salient regarding malaria programmes introduced in resource-constrained countries^34^. Indeed, there has been an exceptional increase recently in funding to decrease malaria transmission worldwide^35^. About 35 countries are now on the path to eliminate malaria, and reinvigorated control interventions are taking place in the remaining endemic countries to envision elimination in the near future^3,36,37^. With this renewed attention and effort, it is essential to evaluate the health impacts attributable to routinely implemented national programmes^38–40^. We argue that, even if they were mainly developed and used in econometrics^41–44^, endogenous treatment effects analyses can contribute to bridging a widening gap in evaluation methods needed to measure progress towards achieving malaria elimination.

## Methods

### SMC programme in Mali

Malaria is one of the single most important public health issues in Mali. In 2016, about 2,311,000 malaria cases were confirmed out of a total population of 18 million, with an estimated number of deaths attributable to malaria close to 21,000^3^. The latest Malaria Indicator Surveys (MIS) revealed a parasitaemia prevalence detected by RDT of 32% in children 6–59 months during the high transmission season, and a prevalence of moderate to severe anemia of 63%^22^. In 2013, the WHO estimated that Mali was the country with the highest malaria incidence in the world, with 460.9 cases per 1000 persons at risk^45^.

Malaria transmission is highly seasonal in Mali. In 52 of the 60 health districts, >60% of the annual rainfall occurs within three consecutive months (July, August and September)^12,46^, which is the threshold used by WHO to recommend SMC in an area^47^. Because of this epidemiological profile, and based on WHO recommendations, Mali introduced SMC as a pilot project in 2012 and progressively expanded it to 48 health districts in 2015^22^. Unfortunately, data on the precise population coverage within these districts is not available.

In Mali, SMC is coupled with a test and treat strategy. In the presence of fever or history of recent sickness, or upon request by the parents, children are tested for malaria using RDTs. If the RDT is positive, children receive malaria treatment (ACTs) and are excluded from SMC. If the RDT is negative, they do not receive ACTs and can be included in the SMC campaign. The regimen used for SMC combines one dose of SP and three daily doses of AQ. This treatment is still effective in Mali, where the prevalence of markers of resistance to SP and AQ is low^16,48^. All children 3–59 months are eligible to receive SMC unless they received ACTs, are febrile, allergic to SP or AQ, or under HIV treatment. The first dose of treatment is administered by health workers at fixed locations in villages. The two remaining doses of AQ are given to the caregiver to be administered at home. Treatments administered are recorded on an SMC card that is given to all children at the first cycle of distribution. Four cycles are organized each year, one per month between August and November.

### Data source

Data used for this study come from the MIS conducted in Mali from September to November, 2015. The MIS is a nationally representative survey, but for security reasons, the three northern regions (Gao, Timbuktu and Kidal) were excluded from the sampling frame. Households were selected using a two-stage cluster sampling approach. First, 177 enumeration areas were selected based on probability proportional to size. Then, 24 households per enumeration area were randomly selected, for a total of 4,243 households identified and 4,240 that were actually surveyed (overall participation rate of 99.9%).

Fieldworkers administered a standardized questionnaire developed by the Demographic and Health Surveys Programme and approved by Roll Back Malaria that included questions at the household and individual levels. All women 15–49 years of age were eligible for the individual section of the questionnaire. At the household level, the MIS collects data on the household’s composition, socio-economic characteristics, and possession of bed nets. At the individual level, the MIS records individuals’ characteristics, malaria-related practices and knowledge, and self-reported exposure to interventions against malaria.

A section specific to children 6–59 months collects data on recent episodes of fever, treatment-seeking behavior, and exposure to SMC. For these children, biological tests are performed using whole blood via finger-prick to detect anemia and malaria parasitaemia. Anaemia is detected by measuring hemoglobin using a HemoCue® portable photometer. A rapid diagnostic test (RDT) for malaria is also performed to detect the presence of pLDH and HRP-2 antigens (SD Bioline Pan/Pf ®). Finally, thick blood smears are prepared in the field, then stained and read by trained microscopists at the Laboratory of Parasitic Diseases, National Institute for Public Health Research in Bamako.

### Design and study variables

We designed a post-test only with non-randomized control group study. The population study consists of all children aged 6–59 months nested in the 4,240 households surveyed. The intervention group includes children who received at least one SMC treatment during the 2015 campaign, while the control group includes children who had not received an SMC treatment at the time of the MIS.

The primary outcome variables are the presence of *Plasmodium* parasites detected by microscopy and the detection of *Plasmodium* antigens (either pLDH or HRP-2) by RDT. Both tests have limited sensitivity and specificity when compared to polymerase chain reaction as the gold standard^23,49^. A common issue with HRP-2 based RDTs is that the antigen can persist in the bloodstream for 4–5 weeks after parasite clearance^50–52^, and therefore the result is more indicative of a period prevalence^53,54^. On the other hand, microscopy detects only current infections, but has poor sensitivity, especially in low transmission areas^23,24^. The secondary outcome variable is the presence of a moderate to severe anemia, defined according to the WHO as a hemoglobin concentration under 10 g/dL^28^.

Self-reported exposure to SMC was assessed at the time of the survey. Because of the partly overlapping dates of the survey and the intervention, children were not exposed to the full SMC campaign (i.e. the four cycles). The exposure variable was categorized and defined as having received an SMC treatment at least during one cycle of the 2015 SMC campaign (yes/no). When available, self-reported exposure was cross-validated by reviewing the children’s SMC cards (85% of the exposed children showed a SMC card). Potential confounding factors (as captured by the MIS) were use of bed nets, age and sex of the child, mother’s education, the household’s size, region, ethnic group, religion, number of bed nets, socio-economic status (SES), and the cluster altitude^33,55,56^. We categorized mothers’ education as none, primary, or secondary and higher, and size of the household as 1 to 5, 6 to 10, or 11 and more. Quintiles of SES were constructed using principal component analysis based on a list of household assets and physical characteristics^57,58^. A malaria infection detected by RDT or microscopy was examined as a potential mediator between exposure to SMC and anemia.

### Statistical analyses

The biggest threat to internal validity with post-test only designs comes from selection bias, i.e., the possibility that observed differences in outcomes between the exposed and control groups reflect differences in their histories or characteristics, rather than being attributable to the intervention^21^. Multiple regression modeling was used to control for observed confounding factors. However, unobserved heterogeneity may also be correlated to both the outcome and the probability of receiving SMC – a situation in which the treatment is endogenous^44^. To mitigate such selection bias on unobservables, we used a treatment effects approach by adding a latent variable related to the likelihood of receiving SMC and the outcome^43,59^. All analyses were performed using Stata® v14.

Based on the Rubin-Neyman potential outcome framework, we used a generalized structural equation model (GSEM) to assess the effects of SMC on malaria parasitaemia and on anemia^60^. This allowed us to simultaneously model the exposure and the outcome (treatment effects), and to measure direct and indirect effects of SMC^61^. Indeed, RDT-confirmed malaria was considered as a potential mediator between SMC treatment and anemia. The working hypotheses are that: (i) by reducing the risk of having been recently infected with malaria, SMC indirectly reduces the risk of presenting a moderate to severe anemia, and that (ii) SMC has no direct effect, positive or negative, on anemia (placebo test on fake outcome^20^).

Therefore, three multiple regression equations were simultaneously computed, whose outcomes are: (i) exposure to SMC, (ii) result from malaria tests, and (iii) presence of moderate to severe anemia. All observed potential confounding factors were entered in the three equations. Parsimony was achieved through the backward stepwise method; variables that did not alter the main coefficient by 5% or more were gradually removed, and excluded variables were re-tested in the final model. Akaike information criterion (AIC) and Bayesian information criterion (BIC) were used to inform model selection^62^.

For each equation, the Poisson distribution with robust variance estimators was preferred over the logistic distribution to obtain model-based risk ratio estimates, as recommended in the literature on impact assessment^63–65^. The linearity of the relationship between each outcome and continuous covariate was assessed by adding quadratic terms. Multicollinearity was ruled out by using the collin package (STATA, College Station, Texas) and verifying that variance inflation factors did not exceed 4. SMC indirect effects were calculated using the product-of-coefficients method, which is standard procedure for Log-link functions^17,61^.

GSEM is an exceptional tool for causal mediation analysis, but with strong assumptions and few tests, if any, to assess goodness-of-fit^66^. Sensitivity analysis was therefore conducted by fitting a Poisson regression model that includes an endogenous binary treatment variable. Estimates obtained from the GSEM and from the endogenous treatment-effects model were compared, and goodness-of-fit assessment was performed on the latter. Children who had received an artemisinin-combination therapy in the two weeks prior to the survey were excluded from the analyses. The threshold for statistical significance was set at 0.05 (bilateral tests).

### Ethics

All survey protocols, questionnaires, and datasets are publicly available online. The protocol for the 2015 MIS received approval from the Research Ethics Committee of the National Institute for Public Health Research in Mali. All methods were performed in accordance with the relevant guidelines and regulations. Informed consent was obtained from all the participants as described in the protocol. Approval to use the MIS data was obtained from the Demographic and Health Surveys Programme.
